# Bifunctional Pd‐Pt Supported Nanoparticles for the Mild Hydrodeoxygenation and Oxidation of Biomass‐Derived Compounds

**DOI:** 10.1002/cssc.202402641

**Published:** 2025-01-17

**Authors:** Vincenzo Ruta, Luis A. Cipriano, Giovanni Di Liberto, Robert Wojcieszak, Gianvito Vilé

**Affiliations:** ^1^ Department of Chemistry Materials and Chemical Engineering “Giulio Natta” Politecnico di Milano Piazza Leonardo da Vinci 32 IT-20133 Milano Italy; ^2^ Department of Material Sciences University of Milan Bicocca Via R. Cozzi 55 IT-20125 Milano Italy; ^3^ Laboratoire Lorraine de Chimie Moléculaire – L2CM UMR 7053 Université de Lorraine and Centre National de la Recherche Scientifique (CNRS) F-54500 Vandœuvre-lès-Nancy France

**Keywords:** Bimetallic nanoparticles, biomass conversion, hydrodeoxygenation reactions, catalyst design, heterogeneous catalysis

## Abstract

The conversion of bio‐based molecules into valuable chemicals is essential for advancing sustainable processes and addressing global resource challenges. However, conventional catalytic methods often demand harsh conditions and suffer from low product selectivity. This study introduces a series of bifunctional Pd_*x*_Pt_*y*_ catalysts supported on TiO_2_, designed for achieving selective and mild‐temperature catalysis in biomass conversion. Synthesized *via* a sol immobilization method and characterized by XRF, N_2_ physisorption, HRTEM, HAADF‐STEM, and XPS, these catalysts demonstrate superior selectivity and activity over monometallic counterparts. In fact, at 20 bar H_2_, Pt/TiO_2_ show a low selectivity in benzophenone hydrodeoxygenation, favoring the benzhydrol hydrogenation product; similarly, Pd/TiO_2_ preferentially form the diphenylmethane hydrodeoxygenation (HDO) product, but with slow conversion rates. The synergistic combination of the two metals in Pd_4_Pt_1_/TiO_2_ drastically improve performance, with 100 % benzophenone conversion and 73 % diphenylmethane selectivity. DFT calculations confirm the synergy between Pd and Pt as the key to drive the activity and selectivity. Additionally, the catalysts also demonstrate high recyclability with minimal performance loss, and have been generalized for the HDO of vanillin and furfural, and in HMF oxidation. Overall, this work highlights the potential of bimetallic catalysts in enabling efficient and selective bio‐based molecule conversion under mild conditions.

## Graphical abstract

This study investigates bimetallic Pd_*x*_Pt_*y*_/TiO_2_ catalysts for the hydrodeoxygenation and oxidation of bio‐derived building blocks at mild conditions, bypassing the need for extreme temperatures typically required in these transformations. The results reveal that tuning the metal ratio is key to activating substrates at low temperatures and achieving high conversion and selectivity in both transformations.

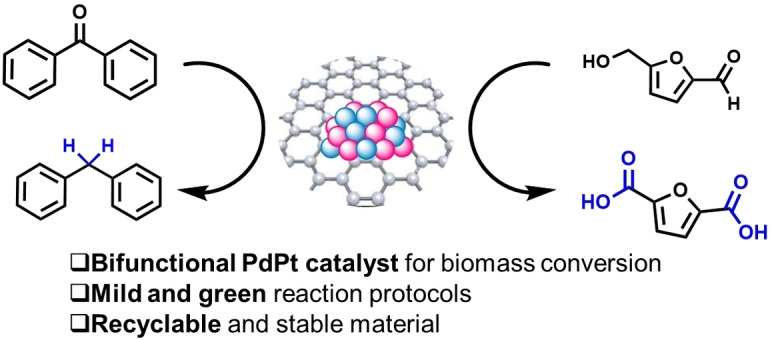

## Introduction

**Figure 1:**
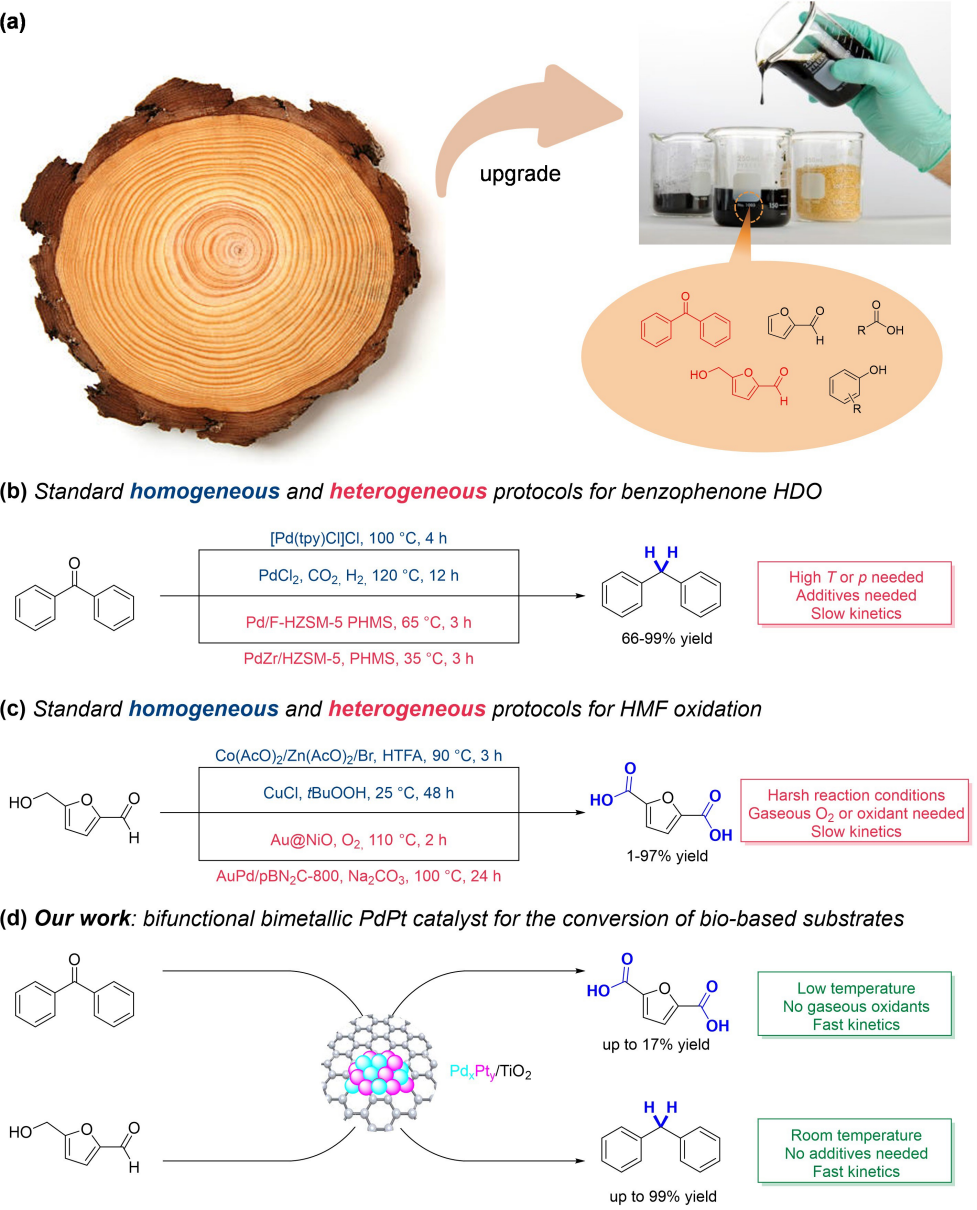
Schematic representation of lignin catalytic upgrading (a); state‐of‐the‐art for benzophenone HDO reactions (b) and HMF oxidation (c), with the advantages and disadvantages when compared to this work (d). An in‐depth comparative analysis with the literature is included in the Supporting Information and underlines the effectiveness of our protocol, which enables similar product yield under milder conditions, *i. e*., in the absence of gaseous oxidants, using lower reaction temperatures, and shorter reaction times, compared to previous protocols.

The pressing demand for the sustainable production of chemicals and fuels from renewable feedstocks has fostered advancements in catalytic methods for the upgrading of biomass‐derived compounds.[^1^, ^2^] In this context, hydrodeoxygenation (HDO) reactions have emerged as critical processes for converting oxygen‐rich biomass into valuable fuels and chemicals,[^3^, ^4^, ^5^, ^6^, ^7^] and extensive research has focused on converting lignin and other biomass‐derived substrates and generate a variety of aromatic and/or aliphatic hydrocarbons.[^6^, ^7^, ^8^, ^9^] From a catalytic viewpoint, significant progress has been made in the development of molecular catalysts (*i. e*., phosphonium salts,^[8]^ or Ru complexes)^[12]^ which often require harsh reaction conditions in terms of time and temperature, as well as the need for H‐transfer additives (*e. g*., *p*TsOH,^[8]^ sylanes, or Et_3_SiH)^[12]^ as hydrogen source, leading to complex downstream operations and significant challenges in catalyst recycling.^[13]^ To bypass these issues, a promising strategy has involved the heterogenization of metal components through deposition of metal nanoparticles onto various heterogeneous supports.[^14^, ^15^, ^16^, ^17^] These catalysts often consist of monometallic nanoparticles based on transition metals (*e. g*., Co,^[18]^ Pd,^[19]^ Ni,^[20]^ and Ru)^[21]^ supported on carbonaceous polymeric materials (*e. g*., carbon,^[22]^ or N‐doped carbon)^[18]^ or inorganic carriers (*e. g*., zeolites^[23]^ or SiO_2_).^[24]^ Despite the increased recyclability and stability of these heterogeneous catalysts,^[25]^ there remains room for further enhancements in HDO reaction protocols, particularly regarding the development of catalytic protocols featuring milder reaction temperature, shorter duration, and higher product selectivity (Figure 1).^[26]^

Similarly, 5‐hydroxymethylfurfural (HMF) has been extensively studied due to its versatility in various transformations, *i. e*. hydrogenation,[^21^, ^22^, ^23^, ^24^, ^25^] HDO,^[30]^ esterification,^[31]^ etherification,^[32]^ reductive amination.^[33]^ In particular, the catalytic oxidation of HMF to 2,5‐furandicarboxylic acid (FDCA) has gained increasing attention,[^34^, ^35^] due to the possibility of using the dicarboxylic acid to replace terephthalic acid in the production of polyethylene terephthalate.[^36^, ^37^] Also in this case, established catalytic protocols often involve harsh reaction conditions, such as gaseous oxidants (air,^[38]^ O_2_),^[39]^ long reaction times (often up to 100 h),[^40^, ^41^] high pressure (10–40 bar) and high temperatures (up to 150 °C).[^30^, ^31^, ^32^, ^33^, ^34^, ^35^, ^36^, ^37^, ^38^, ^39^, ^40^, ^41^, ^42^] Moreover, all catalysts designed thus far, including molecular catalysts (*e. g*., Co(AcO)_2_/Zn(AcO)_2_/Br^−^,^[43]^ or CuCl),^[44]^ and metal nanoparticles (*e. g*., Au,^[45]^ Pd,^[45]^ and Ru‐based)^[46]^ loaded on heterogeneous supports (*e. g*., Al_2_O_3_,^[47]^ NiO,^[35]^ La_2_O_3_,^[48]^ MnCo_2_O_4_,^[49]^ C,^[50]^ or activated C),^[42]^ suffer from low selectivity.

Bimetallic catalysts are gaining attention as a potential solution to enhance the selectivity of bio‐based processes.[^51^, ^52^, ^53^] In this case, the presence of a second metal species modifies the electronic and morphologic structure of the nanoparticles, leading to a synergistic effect that increases the catalyst performance in terms of efficiency and selectivity.[^54^, ^55^, ^56^] In the literature, only a few examples of bimetallic catalysts have been reported that are able to perform both hydrodeoxygenation and oxidation,[^6^, ^57^, ^58^] and those reported lack an in‐depth rationalization of the effect of the bimetallic nature of the material. This emphasizes the necessity of rationally designing new and highly efficient bimetallic catalysts tailored for these applications.

We present herein a series of bifunctional Pd_*x*_Pt_*y*_ catalysts supported on TiO_2_ for the HDO reaction of biomass‐related carbonylic molecules. The study encompasses a comprehensive structural and catalytic investigation of the synthesized materials, aiming to establish a precise structure‐activity relationship attributed to the bimetallic nanoparticle configuration, to modulate selectivity towards the desired final products. To support the experimental data, we also carried out DFT calculations by considering the adsorption of model functional groups on monometallic and bimetallic surfaces of palladium and platinum. The calculations enabled us to elucidate the effect of the bimetallic structure in the reaction. Overall, the work demonstrates that, through the design of a bimetallic system, bio‐based reactions can occur at mild temperatures, in stark contrast with existing methods that operate under harsh conditions..

## Results and Discussion

### Catalyst Synthesis and Characterization

**Figure 2:**
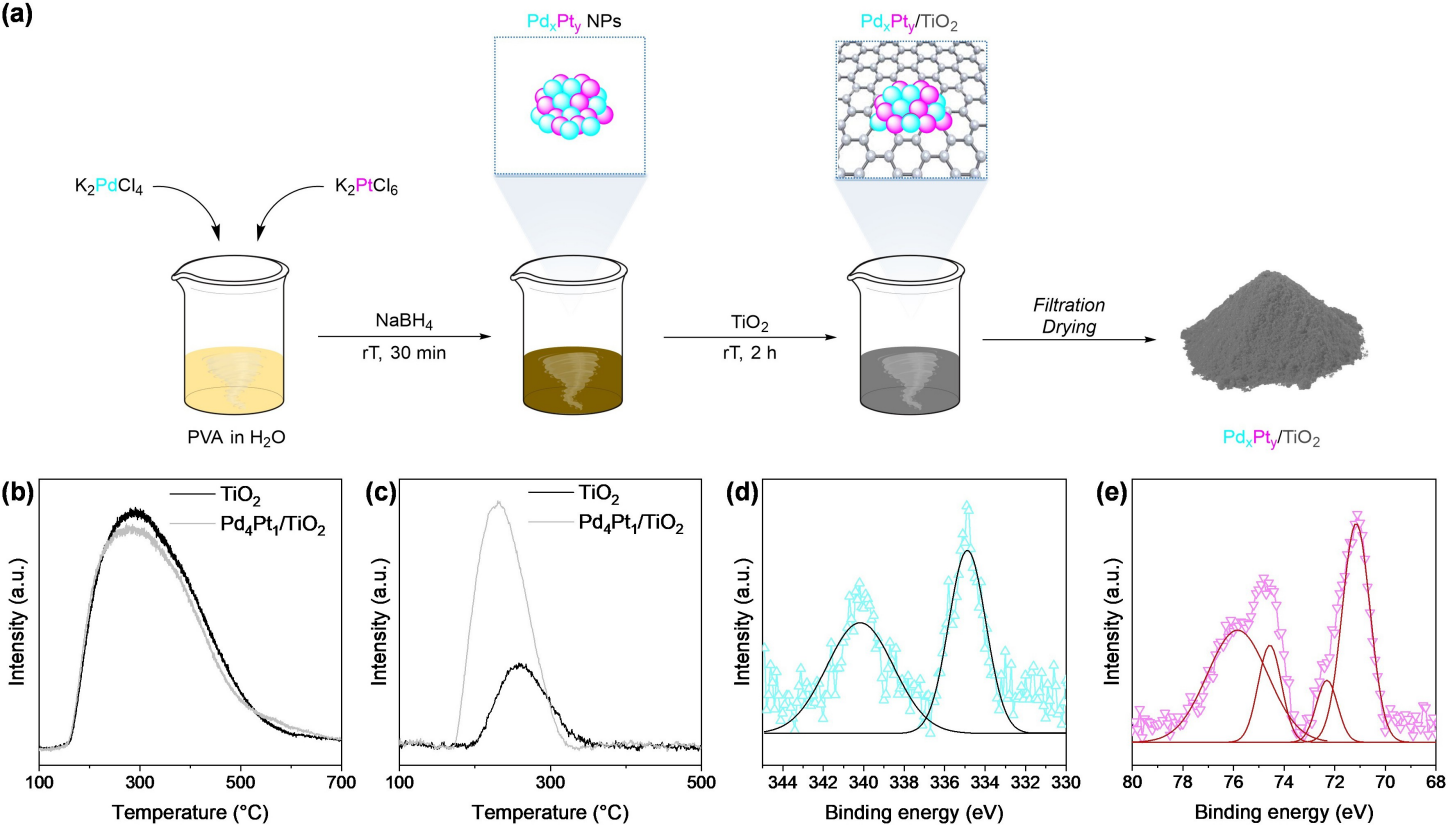
Schematic representation of the synthesis of the bimetallic Pd_*x*_Pt_*y*_/TiO_2_ catalysts (a); NH_3_‐TPD (b) and CO_2_‐TPD (c) analyses of TiO_2_ and Pd_*x*_Pt_*y*_/TiO_2_ materials; Pd 3*d* (d) and Pt 4*f* (e) XPS core level spectra of the bimetallic Pd_*x*_Pt_*y*_/TiO_2_ catalyst. The characterizations showed in the figure refer to Pd_4_Pt_1_/TiO_2_.

The preparation of the bimetallic Pd_*x*_Pt_*y*_/TiO_2_ catalyst series was performed *via* sol immobilization technique,[^59^, ^60^] and the synthetic procedure is detailed in the Supporting Information and sketched in Figure 2a. At first, colloidal metal nanoparticles were formed by mixing Pd and Pt precursors, namely K_2_PdCl_4_ and K_2_PtCl_6_, with poly(vinyl)alcohol (PVA) as a stabilizing agent to control the nanoparticle size.^[61]^ Specifically, low molecular weight PVA was chosen from various stabilizers because it could be easily removed by washing the catalyst with water at the end of the synthetic procedure. Subsequently, the colloidal nanoparticles were reduced using NaBH_4_ and anchored onto the TiO_2_ support through impregnation with stirring at room temperature for 2 h. Within this technique, we synthesized bimetallic materials with varying Pd : Pt mass ratios (1 : 4, 1 : 1, 4 : 1), along with monometallic Pd/TiO_2_ and Pt/TiO_2_. Prior to catalytic evaluation, these mono‐ and bimetallic catalysts were extensively characterized. Metal loading onto the support and Pt : Pd ratio on the different catalysts was confirmed by X‐ray fluorescence (XRF) analysis, as reported in Table 1, proving the efficiency of the deposition method.

**Table 1:** Structural and textural analysis of monometallic and bimetallic Pd_*x*_Pt_*y*_/TiO_2_ catalysts.

Entry	Catalyst	Pt^*[a]*^ (wt. %)	Pd^*[a]*^ (wt. %)	*S*_BET_^*[b]*^ (m^2^ g^−1^)	*V*_pore_^*[c]*^ (cm^3^ g^−1^)	Acid sites^*[d]*^ (mmol g^−1^)	Basic sites^*[e]*^ (mmol g^−1^)
**1**	TiO_2_	–	–	54	0.113	0.26	0.01
**2**	Pt/TiO_2_	1.33	–	54	0.115	0.24	0.03
**3**	Pd/TiO_2_	–	1.58	56	0.129	0.23	0.02
**4**	Pd_4_Pt_1_/TiO_2_	0.30	1.21	49	0.143	0.25	0.03
**5**	Pd_1_Pt_1_/TiO_2_	0.74	0.75	50	0.113	–	–
**6**	Pd_1_Pt_4_/TiO_2_	1.04	0.26	45	0.114	–	–

*[a]* XRF data; *[b]* BET surface area from the N_2_ isotherms collected at −196 °C; *[c] V*_pore_ was determined from the amount of N_2_ adsorbed at *p/p_0_* = 0.99. *[d]* NH_3_‐TPD data; *[e]* CO_2_‐TPD data.

**Figure 3:**
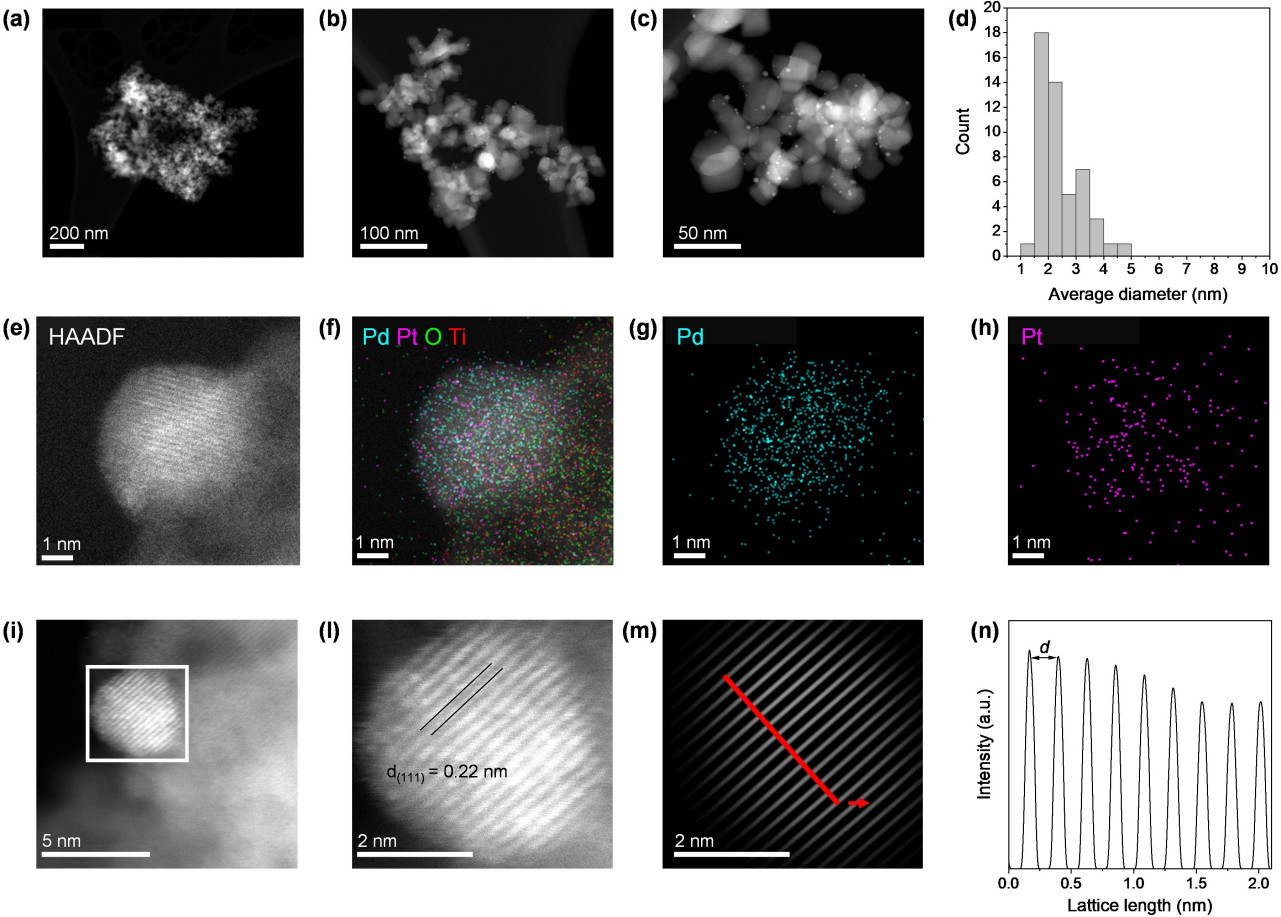
TEM of the bare support (a); HRTEM of the produced catalysts at two different magnification (100 and 50 nm, b and c), and related particle size distribution (d); HAADF‐HRTEM of the bimetallic nanoparticles (e), with EDS mapping (f, g, h); studies for the *d*‐spacing calculation from the crystal lattice for bimetallic nanoparticles (i, l), carried out *via* FFT‐reverse FFT process (m, n).

Textural properties were determined *via* N_2_ physisorption experiments (Table 1, Figure 2b, and Figure S1, Supporting Information), finding that the deposition of bimetallic nanoparticles did not reduce the surface area with respect to the bare TiO_2_ support (54 m^2^ g^−1^). This is likely due to the small dimension of the nanoparticles (*vide infra* for particle size distribution and micrographs), combined with the low metal loading. Pore volume analysis and pore size distribution (Table 1 and inset of Figure S1a, Supporting Information) confirmed the non‐porous nature of both monometallic and bimetallic materials. Additionally, no significant changes in these properties were observed upon metal incorporation. The morphology and particle size of the catalytic material were evaluated *via* high‐resolution transmission electron microscopy (HRTEM). The micrographs of the bimetallic catalysts (Figure 3a, 3b, and 3c) showed the even distribution of the nanoparticles throughout the whole surface of the support. The histogram of the particle size distribution (Figure 3d) shows that the particles are mainly distributed in the diameter between 1.5 and 3.5 nm; thus, the size distribution can be fitted approximately by a Gaussian function centered at 2.2 nm, with only a few nanoparticles bigger than 4 nm. To verify the alloyed nature of the metals in the nanoparticle structure, we performed HAADF‐EDS elemental mapping (Figure 3e–h). Elemental mapping obtained *via* EDX analysis provided direct evidence of the intimate contact between Pd and Pt within the same nanoparticles, as shown in the single‐particle micrographs. This co‐localization indicates strong interactions between the two metals, ruling out the possibility of segregated clusters or isolated nanoparticles. Moreover, analysis of the lattice pattern of several different nanoparticles located in multiple regions across the sample was perfromed through fast Fourier transform (FFT) and inverse FFT of HAADF‐STEM images. These studies, as illustrated in Figure 3i–n and Figure S2, Supporting Information, revealed a major number of nanoparticles presenting a *d*‐spacing of 0.22 nm, where this value is characteristic of the (111) facet of bimetallic PdPt nanoparticles. In addition, a minor number of (200) facets was also detected, mainly located in corners and edges of the nanoclusters. The (200) facet is well known to be harmonics of parallel crystallographic plane (100), thus we can study as (100) planes with high reliability. Additional pieces of information were obtained by performing CO_2_ and NH_3_ temperature‐programmed desorption analyses (CO_2_‐TPD and NH_3_‐TPD), allowing the titration of, respectively, basic and acidic sites.^[62]^ NH_3_ desorption (Figure 2b) on both the catalysts and the metal‐free support showed a single, large peak ranging from 190 to 500 °C, proving the abundant presence of weak acidic sites, although the concentration of adsorbed NH_3_ slightly decreased in the nanoparticles‐loaded catalysts (0.23–0.25 mmol g^−1^ in both monometallic and bimetallic samples, with respect of 0.26 mmol g^−1^ in the bare support). Regarding CO_2_ desorption analysis (Figure 2c), TiO_2_ presented a low concentration of basic sites producing a peak ranging from 200 to 300 °C, while it is evident a metal‐induced increased CO_2_ desorption in the metal‐loaded catalysts, leading to a broadening of the peak at 200 °C (*ca*. 0.02–0.03 mmol g^−1^ of CO_2_ desorbed, with respect of 0.01 mmol g^−1^ of the bare support). Furthermore, the comparison between the metal‐loaded catalysts and the bare support revealed that the incorporation of such small metal nanoparticles did not generate additional peaks but instead altered the intensity of those associated with the support, highlighting that the basic sites originate from the support itself. Moreover, both monometallic and bimetallic materials showcased similar values for the quantification of acid and basic sites, underlying that the alloyed nature of the bimetallic nanoparticles is not altering these properties, with respect of monometallic ones. Finally, XPS analysis was performed to assess the oxidation state of the metals in the catalyst. Particularly, the Pd 3*d*_*5/2*_ core level spectrum (Figure 2d) revealed the characteristic peak of Pd^0^ centered at 335.97 eV and its typical satellite peak at 340.12 eV. Indeed, the Pt 4*f*_*7/2*_ spectrum (Figure 2e) clearly shows a principal peak that can be deconvoluted into two peaks centered at 71.1 eV and 72.25 eV, indicating the presence of both Pt^0^ and Pt^+^. Even in this case, a satellite peak can be observed, centered at 75.4 eV.^[63]^ Overall, the comprehensive characterization demonstrated the successful synthesis of bimetallic Pd_*x*_Pt_*y*_/TiO_2_ catalysts (together with their monometallic counterparts) with finely dispersed nanoparticles on TiO_2_.

### Benzophenone Hydrodeoxygenation over Pd_*x*_Pt_*y*_ Catalysts

**Figure 4:**
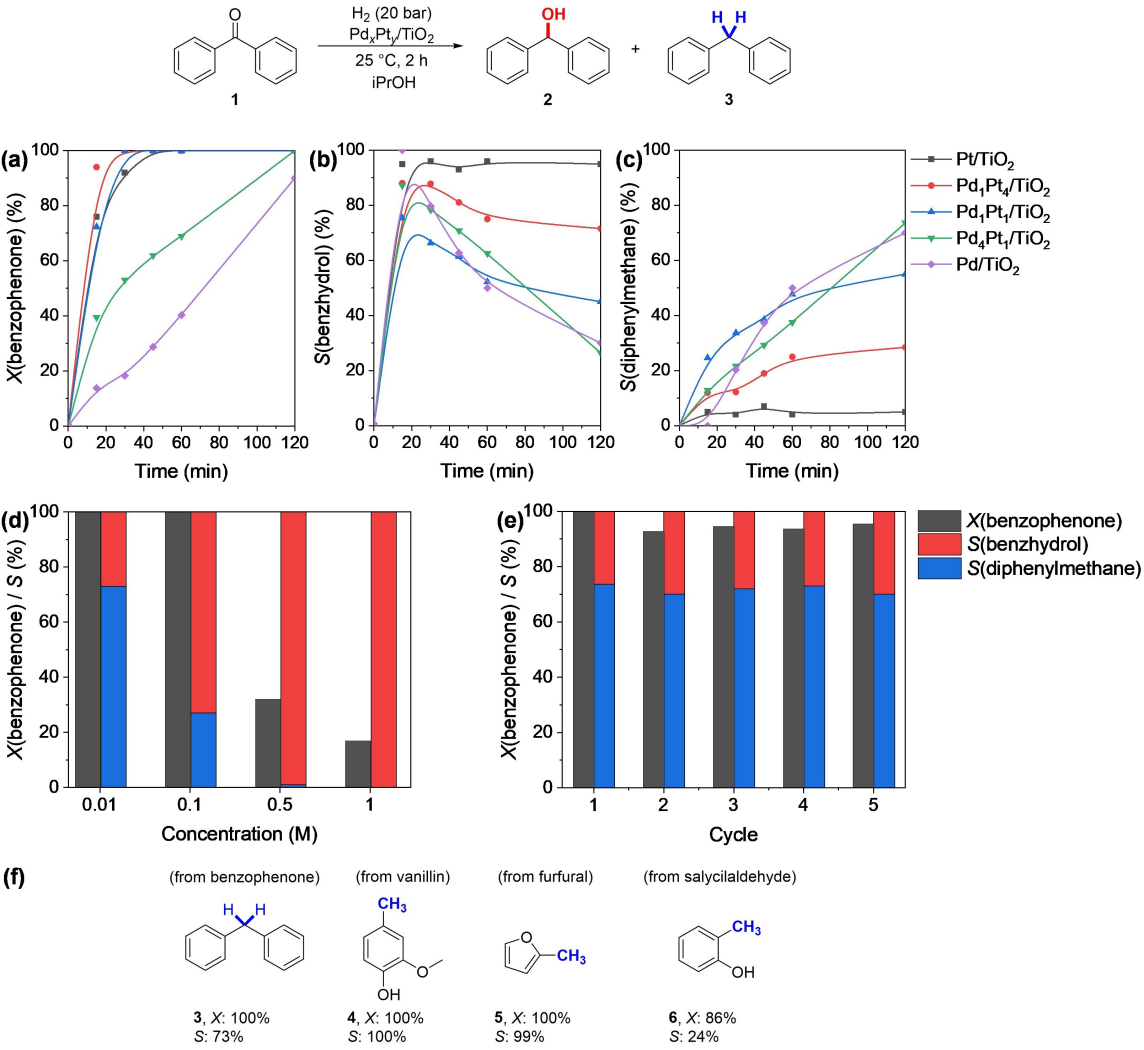
Catalytic tests for the hydrodeoxygenation of biomass‐related compounds. Kinetic profile of conversion of benzophenone 1 (a), selectivity for the hydrogenation intermediate 2 (b), and for the HDO product 3 (c); reactant concentration screening (d) and recyclability test over 5 reaction cycles (e) for the benzophenone HDO; screening of different biomass‐based aldehydes and ketones HDO (e). Reaction conditions: substrate (0.15 mmol) and catalyst (50 mg) in iPrOH (10 mL), H_2_ (20 bar), for 2 h at 25 °C; for (d), (e), and (f) the catalyst used is Pd_4_Pt_1_/TiO_2_.

The aforementioned catalysts were first tested in the hydrodeoxygenation of benzophenone, a model compound commonly present in the bio‐oils obtained from the catalytic upgrading of lignocellulosic biomass.[^64^, ^65^] We started our investigation with a series of control experiments reported in Table S1, Supporting Information. Particularly, the lack of product formation in the reaction without the catalyst confirmed that the reaction is truly catalytic; similarly, conducting the test in the presence of bare TiO_2_ resulted in no conversion of the starting material, which evidenced the importance of the metal for the HDO reaction. The same outcome was observed when conducting the reaction under N_2_ pressure, thus excluding the possibility of transfer hydrogenation mediated by the solvent. The results demonstrated that the reaction required a metal catalyst for successful hydrogenation, and the solvent type was not critical for the process. After these controls, the impact of the bimetallic nature of the catalytic materials, as well as the effect of the metal ratio was elucidated. The conversion and selectivity profiles obtained are depicted in Figure 4a and b. All catalytic tests were carried out at room temperature, for 2 h, in an H_2_ atmosphere (20 bar), with the absence of external additives to keep the protocol greener. Microkinetic data obtained from the tests with monometallic catalysts (Pd/TiO_2_ and Pt/TiO_2_) led to some tentative mechanistic insights on the reaction: specifically, the detection in the early stages of benzhydrol **2** (Figure 4), whose concentration decreases in time, in parallel with the increase of concentration of diphenylmethane **3**, proved that the reaction occurred in two separate and consecutive steps, namely (i) the hydrogenation of the carbonylic C=O bond, affording the secondary alcohol **2**, and (ii) the hydrogenolysis of the C−OH bond, with the formation of the final product **3** and H_2_O. Moreover, the outcome of these tests revealed the role of the two metals in the reaction mechanism: Pt/TiO_2_ led to the complete conversion of benzophenone **1**, with high selectivity for benzhydrol **2** (95 %) and low detection of diphenylmethane **3**, indicating that Pt is mainly involved in the hydrogenation step. On the other hand, the test of Pd/TiO_2_ resulted in a slightly minor and slower conversion of **1** (90 %), but an increased selectivity for the HDO product **3** (70 %), revealing the main involvement of Pd in the HDO reaction. Therefore, the design of the series of bimetallic catalysts with alloyed Pd_*x*_Pt_*y*_ nanoparticles could effectively tune the reaction selectivity through the synergistic effect of the two metals. This hypothesis was confirmed by testing the three bimetallic catalysts Pd_*x*_Pt_*y*_/TiO_2_, with an enhancement in all cases for the selectivity of **3** when compared with Pt/TiO_2_, and a higher conversion of starting material **1** when compared to Pd/TiO_2_. This improved performance could be attributed to the synergistic effect of the coexistent presence of both metals in the alloyed nanoparticles, while nanoparticles composed of a larger content of Pt led to diminished reaction performances in terms of selectivity for the HDO product **3**, as suggested by data for Pd_1_Pt_4_/TiO_2_ and Pd_1_Pt_1_/TiO_2_. On the other hand, the formation of alloyed Pd_*x*_Pt_*y*_ nanoparticles featuring only a minor amount of Pt led to the increase of the hydrogenation rate, as shown by the results over Pd_4_Pt_1_/TiO_2_. Thus, Pd aided in maintaining high selectivity for the HDO product **3**. The outcome of these tests indicated that the synergy of the metal is the only factor responsible for increased catalytic activity, as other relevant material features, namely acid‐base properties, pore size distribution and surface area, present only minor variations between monometallic and bimetallic catalysts. In addition, selectivity control can be efficiently operated by simply varying the Pd : Pt ratio in the bimetallic nanoparticles. Indeed, increasing the Pt content the hydrogenation pathway results favored, while increasing the Pd amount the reaction prosecutes favoring the formation of the HDO product.

We continued our investigation by evaluating the effect of benzophenone concentration in the reactant solution, using thus Pd_4_Pt_1_/TiO_2_ as a catalyst. The outcome of this study, depicted in Figure 4c, showed a linear decrease in selectivity for HDO product **3** with the concentration increase at 0.1 M (27 %) while retaining the total conversion of benzophenone **1**. A further increase in reactant concentration at 0.5 and 1 M led to a dramatic decrease also of the conversion (respectively 32 % and 17 %), while the only product detected was benzhydrol **2**. Catalyst stability and recyclability were evaluated with a recycling test (Figure 4d). The results stressed the steady performance of the catalytic system and its stability for five consecutive cycles. Post‐catalytic characterization of the spent catalyst after recycling tests was also carried out, in order to exclude potential modifications of the catalyst structure. TEM showed that, after HDO reactions, the morphology and particle size distributions of the metal nanoparticles remained unaltered, and no segregation of the metallic phase was observed, as evidenced in the micrographs reported in Figure S3, Supporting Information. Moreover, in the XPS profiles, the main peaks at 71.1 eV, 72.25 eV (Pt 4*f*), and 335.97 eV (Pd 3*d*), with related satellite peaks, remained visible in the used catalysts, indicating no major variation of the oxidation state of the metals after reaction (Figure S4, Supporting Information). A decrease in the intensity of Pt signals was also observed in post‐catalysis specimen spectra, which can be primarily attributed to the presence of carbon‐based adsorbates on the used catalyst. This hypothesis was supported by ruling out metal leaching as a potential cause: in this case, inductively coupled plasma optical emission spectroscopy (ICP‐OES) analysis of the post‐catalysis material and final reaction mixture confirmed the absence of any metal leaching.

To broaden the scope of our work, we tested other bio‐based substrates (*i. e*. vanillin, furfural, and salicylaldehyde) with the optimal catalyst Pd_4_Pt_1_/TiO_2_, and the results are shown in Figure 4e. Vanillin and furfural afforded quantitatively the respective HDO products **4** and **5**. Using salicylaldehyde as substrate led also to the production of the HDO product **6**, although with a lower selectivity due to the alcoholic intermediate stabilization *via* intramolecular hydrogen bonding. Nonetheless, the reactivity of these substrates underlined the versatility of the developed catalytic protocol for the mild and efficient hydrodeoxygenation of bio‐based substrates into value‐added chemicals.

### Molecular Understanding of the Selectivity Patterns

**Figure 5:**
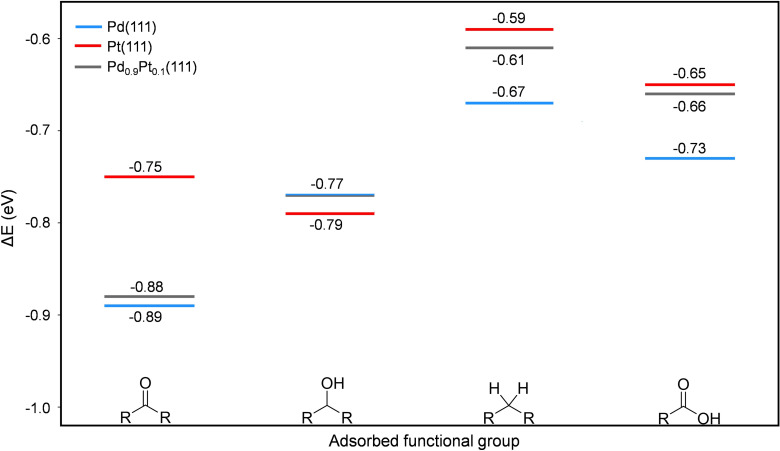
Adsorption binding energies for each functional group on Pd(111), Pt(111), and Pd_0.9_Pt_0.1_(111) surfaces for monometallic and bimetallic catalysts. From left to right: carbonyl, hydroxyl, alkene, and carboxyl functional groups.

To gain an atomistic understanding of the performances of our monometallic and bimetallic systems, density functional theory (DFT) calculations were performed on the two most commonly exposed facets of Pd, Pt, and PdPt nanoparticles, namely the (111) and (100) planes.[^66^, ^67^] Simulating these two surfaces is crucial due to their different physical and chemical properties, and this allows for a better understanding of their distinct catalytic behaviors, which can aid in optimizing catalyst design for a specific reaction. The DFT simulations were performed with the Perdew‐Burke‐Ernzerhof (PBE)^[68]^ functional including dispersion forces, as implemented in the Grimme′s D3 scheme,^[69]^ which represents a reasonable choice when studying adsorption on metal surfaces.[^70^, ^71^, ^72^] Additional details related to DFT and computational details are reported in Figures S5–S10 and Tables S2–S7 of the Supporting Information. These calculations involved the adsorption of various functional molecules; specifically acetic acid to represent a carboxylic acid group, acetone to represent a carbonyl group, ethanol to represent a hydroxyl group, and propane to represent an alkane group. The surface models Pd(111), Pt(111), Pd(100), and Pt(100) described the reactivity of pristine metal surfaces. In the case of the alloyed systems, we simulated Pd_0.9_Pt_0.1_(111) and Pd_0.87_Pt_0.13_(100) models having both Pt and Pd atoms on the topmost layer. The composition is similar to that of the experimental sample, and it was generated to have a good compromise between the reliability of the results and computational cost. The binding energies (Δ*E*) for each functional group on the different surfaces were calculated with respect to the energy of the free molecular species and the surfaces within the following equation (Eq. 1:
(1)

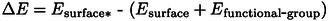




where *E*_surface*_, *E*_surface_, and *E*_functional‐group_ correspond to the energy of the adsorbed functional group on the surface, the “free” surface energy for each catalyst, and the energy of the free functional group. The binding energies for the most stable adsorption species for each functional group are reported in Figure 5 for the (111) surfaces, and in Figure S11, Supporting Information, for the (100) ones.

The results show that the surfaces exhibit different adsorption trends which can be associated with variations in atom density, coordination, and electronic structure. For example, the (111) facet is known to have higher coordination number of the surface metal atoms and for being more stable and selective in reactions such as hydrogenation and oxidation reactions, while the (100) exhibits different surface reactivity because of its less dense atomic arrangement and lower coordination numbers.

To describe the conversion of ketone **1**, we examined the carbonyl group adsorption on the surfaces mentioned previously. Our findings for the (111) facet indicate that this functional group is better adsorbed on the Pd(111) and Pd_0.9_Pt_0.1_(111), with nearly identical Δ*E* rather than on the pure Pt(111) surface. Conversely, the results with the (100) facet indicate that pure Pt(100) is more reactive than Pd(100) and the alloyed Pd_0.87_Pt_0.13_(100) surface, which remains less reactive given the higher presence of Pd. These results illustrate how the different reactivity of both surfaces can lead to different pathways.

Next, we simulated the adsorption of the first product that can be formed during the HDO reaction, *i. e*., hydroxyl functional group. We found that this functional group binds more strongly to both the Pt(100) and Pt(111) surfaces (Figure 5 and Figure S11, Supporting Information, red lines), in line with Figure 4. In particular, the highly‐defective (100) coordination sites are more reactive than the (111) in flat surfaces. The fact that Pt surfaces exhibit stronger adsorption suggests that platinum is more reactive in the benzophenone activation and hydroxyl formation, which well explains the experimental benzhydrol **2** formation when platinum is involved (Figure 4b). The second product formed during the HDO reaction is the formation of diphenylmethane **3**. In our DFT calculations, we simulated the adsorption of the propene molecule to the specified surfaces and found that this molecule does not poison the catalyst when pure Pd(100) and both alloyed Pd_0.9_Pt_0.1_(111) and Pd_0.87_Pt_0.13_(100) surfaces are considered, which aligns with the experimental observations where the Pd_0.8_Pt_0.2_ alloys and Pd showed enhanced formation of diphenylmethane **3** (Figure 5 and Figure S11, Supporting Information, blue and grey lines).

### Application in HMF Oxidation

To further broaden our work‘s scope and demonstrate our material‘s applicability for other reactions in the biomass conversion panorama, we decided to test our mono‐ and bimetallic materials in the HMF oxidation to FDCA. We began our investigation with a rapid optimization of reaction conditions in terms of base, solvent, and catalyst amount, and the results are reported in Table S8, Supporting Information. From the base type study, a more alkaline reaction environment, produced using NaOH as a base, led to increased conversion of HMF and subsequent increase of reaction rate (0.34 mmol_prod_ g_cat_^−1^ h^−1^, Table S8, Entry 3), with respect of an equimolar amount of the weaker base K_2_CO_3_ (0.16 mmol_prod_ g_cat_^−1^ h^−1^, Entry 2); moreover, also the Lewis base F^−^ in CaF_2_ led to detrimental reaction performances in terms of both conversion and selectivity (Entry 1). The effect of the solvent type was also investigated, finding out that less polar solvents, such as MeCN (Entry 4), led to minor conversion and no production of FDCA, due to the worst solubility of both base and atmospheric oxygen in the mixture.^[73]^ Indeed, using a protic and more polar solvent, *i. e*. iPrOH (Entry 5), led to complete conversion of HMF, but also to the formation and precipitation of humins, formed by the oxidative polymerization of both starting material and reaction intermediates,^[74]^ suggesting that the increase of the solubility of the reaction intermediates in the organic media led to an overactivity of the system. Finally, the screening of the amount of catalyst (Entry 7 and 8) led to a decrease in the reaction rate increasing the catalyst amount, due to a diminished HMF conversion. With these optimized conditions in hand, we started our investigation on the structural features of the bimetallic catalysts produced in this series.

**Figure 6:**
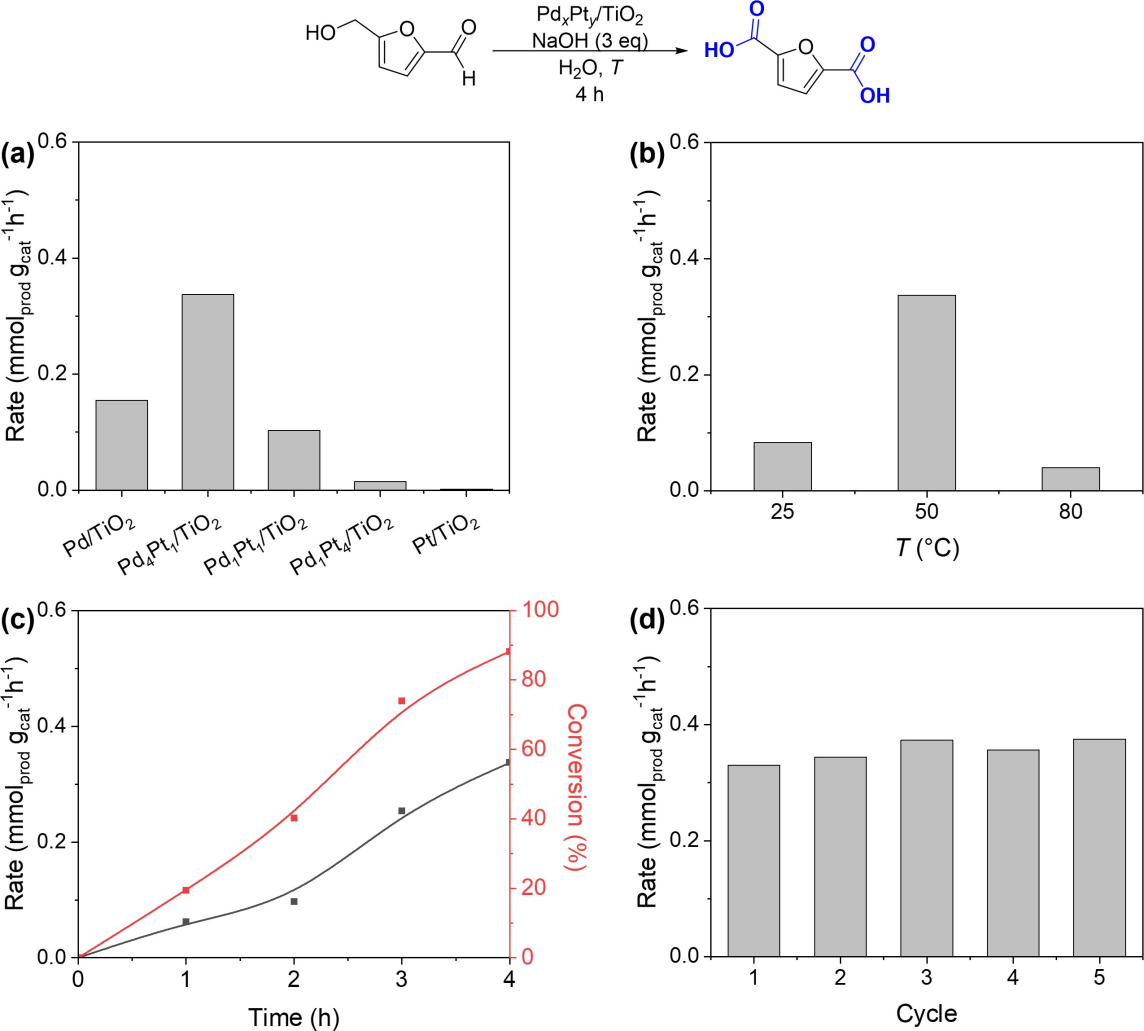
Catalytic tests for HMF oxidation over Pd_x_Pt_y_ catalysts. Different catalysts comparison for the HMF oxidation (a); temperature screening for the HMF oxidation in the presence of Pd_x_Pt_y_ catalyst (b); kinetic studies for the HMF oxidation in the presence of bimetallic catalysts (c); recyclability test over 5 reaction cycle for HMF oxidation (d). Reaction conditions: HMF (0.07 mmol) and catalyst (5 mg) in H_2_O (5 mL), for 4 h, at 50 °C (variable in (b)); for (b), (c), and (d) the catalyst is Pd_4_Pt_1_/TiO_2_.

The outcome of this study is depicted in Figure 6a, which shows the good performance of monometallic Pd‐based catalyst when compared with Pt_1_/TiO_2_. This trend is also maintained in the catalyst Pd_1_Pt_4_/TiO_2_. However, with further minute increases in the amount of Pd it was possible to maximize the yield of FDCA, finding its optimum in the catalyst Pd_4_Pt_1_/TiO_2_. This behavior can be attributed to the synergistic effect related to the coexistence of both metals in the alloyed nanoparticles. Similarly to the case of HDO reaction, these tests confirmed that the bimetallic nature of the catalysts led to superior performances, while other relevant properties (acid‐base properties, surface area, and porosity) remained unaltered in bimetallic and monometallic catalysts. In this case, the insertion of a minimal amount of Pt in the optimal catalyst Pd_4_Pt_1_/TiO_2_ appeared crucial to boost the oxidation rate, enhancing the formation of FDCA as final product, thus not stopping to precedent reactions intermediates. To have a better understanding of the features of our catalytic system, a temperature screening was carried out. An enhancement of the reaction rate was observed by increasing the temperature from 25 to 50 °C. Furtherly increasing the temperature after 80 °C led to minor catalytic improvements in the production of FDCA, as well as the precipitation of humin species was observed. This phenomena is related to the increased solubility of reaction intermediates at higher temperatures, and represents one of the major drawbacks of the HMF oxidation protocols.^[75]^ The reaction kinetics was evaluated by performing regular withdrawal in time from the reaction mixture, elucidating the increase of the catalytic production of the FDCA during the 4 h reaction time, as depicted in Figure 5c. Finally, catalyst recyclability was evaluated through a recycling test (Figure 6d), proving the stability of our bimetallic catalytic material for five reaction cycles. A series of control experiments were performed, and the outcomes are reported in Table S9, Supporting Information. In fact, also in this case, carrying out the reaction in the absence of a catalyst and with bare TiO_2_ led to negligible amount of HMFCA. These experiments evidenced the catalytic nature of our reaction.

The adsorption of a carboxyl group (acetic acid) on the same surfaces described above was simulated with DFT. Our results revealed that the experimental trend where Pd_0.8_Pt_0.2_ alloy is the best catalyst for the HMF reaction (Figure 6a) is well addressed with the (100) facet. This is because the carboxyl group poisoned the Pt(100) surface due to strong adsorption into the surface (Figure S11, Supporting Information, salmon line), while the alloyed Pd_0.87_Pt_0.13_(100) and pure Pd(100) surfaces weakest the adsorption energy of the functional group (Figure S11 Supporting Information, grey line). A strong adsorption hinders the HMF reaction, while weak adsorption allows it. Finally, a comparison with literature precedents (Figure S12, Supporting Information) for the HMF oxidation to afford FDCA allowed us to understand the effectiveness of our protocol, which enables product formation under milder conditions, *i. e*., in the absence of gaseous oxidants, using lower reaction temperature, and shorter reaction time, compared to previous protocols.

## Conclusions

In conclusion, we have developed a series of bimetallic Pd_*x*_Pt_*y*_/TiO_2_ catalysts that efficiently catalyze at low temperature the conversion of bio‐based molecules to obtain value‐added chemicals. The materials were prepared *via* sol‐gel immobilization technique and characterized by means of several analytical techniques, to unveil the structural properties of the materials and the alloyed nature of the supported metal nanoparticles. This series of materials was then tested in the hydrodeoxygenation of different carbonylic compounds and in the oxidation of HMF, demonstrating the bifunctional nature of our catalyst, capable of showing a high performance in both oxidative and reductive reaction environments. The superior catalysis of our system were related to the synergistic action of alloying platinum nanoparticles with palladium as proved in the HDO of carbonylic compounds and HMF oxidation reactions through DFT simulations; moreover, a crucial point in both applications was represented by the possibility of controlling reaction selectivity by easily varying the metal ratio in the alloyed nanoparticles. Overall, this study allowed us to unveil the potential of a bimetallic catalyst for relevant bio‐based substrate conversion reactions, and can be important for the development of greener, milder, and efficient biomass conversion protocols.

## Supporting Information

The data supporting this article have been included as part of the Supporting Information.

## Conflict of Interests

The authors declare no conflict of interest.

1

## Supporting information

As a service to our authors and readers, this journal provides supporting information supplied by the authors. Such materials are peer reviewed and may be re‐organized for online delivery, but are not copy‐edited or typeset. Technical support issues arising from supporting information (other than missing files) should be addressed to the authors.

Supporting Information

## Data Availability

The data that support the findings of this study are available from the corresponding author upon reasonable request.
