# Supplementary material for: Bifunctional Pd‐Pt Supported Nanoparticles for the Mild Hydrodeoxygenation and Oxidation of Biomass‐Derived Compounds
Source: ChemSusChem. 2025 Jan 17;18(9):e202402641. doi: 10.1002/cssc.202402641 (PMC12051223; doi:10.1002/cssc.202402641)
Supplement: Supplementary file 1 — Supporting Information [file CSSC-18-e202402641-s001.pdf]

ChemSusChem

Supporting Information

Bifunctional Pd-Pt Supported Nanoparticles for the Mild Hydrodeoxygenation and Oxidation of Biomass-Derived Compounds

Vincenzo Ruta, Luis A. Cipriano, Giovanni Di Liberto, Robert Wojcieszak,* and Gianvito Vilé*

Supporting Information

Bifunctional Pd-Pt Supported Nanoparticles for the Mild Hydrodeoxygenation and Oxidation of Biomass-Derived Compounds

Vincenzo Ruta,[a] Luis A. Cipriano,[a] Giovanni di Liberto,[b] Robert Wojcieszak,[,c] and Gianvito Vilé*[,a]*

[a] *Department of Chemistry, Materials, and Chemical Engineering "Giulio Natta", Politecnico di Milano, Piazza Leonardo da Vinci 32, 20133 Milano, Italy.*

[b] *Department of Materials Science, Università di Milano Bicocca, via R. Cozzi 55, 20125 Milano, Italy*

[c] *Laboratoire Lorraine de Chimie Moléculaire – L2CM UMR 7053, Université de Lorraine and Centre National de la Recherche Scientifique (CNRS), Vandœuvre-lès-Nancy, F-54500 France.*

* *Corresponding authors. E-mails: robert.wojcieszak@cnrs.fr (R.W.) and gianvito.vile@polimi.it (G.V.).*

Methods

Catalyst preparation. Catalyst preparation was performed *via* sol-gel immobilization technique. A solution of K_2PdCl_4 (Sigma-Aldrich, 99% purity, various amounts) and K_2PtCl_6 (Sigma-Aldrich, 99% purity, various amounts) was prepared in 645 mL of distilled water. Then, PVA (1.2 mL, aq. solution 1 wt.%) was added, and the solution was stirred for 3 min. Colloidal nanoparticle formation was then induced by adding $NaBH_4$ (Sigma Aldrich, 99% purity, 9.6 mg in 2.5 mL of distilled water) to the mixture, stirring at room temperature for 30 min. After this, the TiO_2 (Degussa P25, Evonik, 985 mg) support was added, and the slurry was left under stirring at room temperature for 2 h. At this point, the solution was filtered, and the catalyst was washed with abundant distilled water, then dried in two steps, at room temperature for 12 h, and consequently for 4 h at 80 °C.

Catalyst characterization. X-ray fluorescence (XRF) analysis was performed using a M4 TORNADO (Bruker) spectrometer equipped with 2 anodes, a rhodium X-ray tube 50 kV/600 mA (30 W), and a tungsten X-Ray tube 50 kV/700 mA (35 W); indeed the detector used was a silicon-drift-detector Si(Li) with <145 eV resolution at 100000 cps (Mn Kα), cooled with a Peltier cooling (253°K). The analysis was performed at 20 mbar. Quantitative analysis was done using fundamental parameters (standardless), and for each sample, 36 points (of 200 μm) were analyzed. CO_2 and NH_3 temperature programmed desorption (CO_2- and NH_3-TPD) were carried out using a Micromeritics Autochem II 2920 device. The measurements were carried out by placing powder samples in a U-shaped quartz reactor with an inner diameter of 0.5 cm. The materials were pre-treated under air (30 mL min^{-1}), while being heated from room temperature to 450 °C with a heating rate of 10 °C min^{-1}, the final temperature being kept for 30 min to ensure total elimination of physisorbed water molecules. Then, adsorption of CO_2 or NH_3 (1 vol. % in He) was carried out for 1 h at 100 °C. After that, the samples were purged with a flow of He (50 mL min^{-1}) for 20 min at 25 °C to remove the weakly adsorbed species. Subsequently, TPD was carried out, with a heating rate of 10 °C min^{-1} till 450 °C; this temperature was maintained for 30 min. The desorbed CO_2/NH_3 was analyzed by GC-TCD chromatography and quantified after the calibration curve, expressing the desorption as mmoles of CO_2/NH_3 per gram of catalyst. Nitrogen physisorption measurements were performed after degassing the samples at 150 °C for 15 h in a vacuum using a Micromeritics 3Flex porosimeter at -196 °C. The specific surface areas were calculated by applying the Brunauer-Emmett-Teller (BET) model to adsorption isotherms for

$0.05 < p/p_0 < 0.3$ using the QuadraWin 5.05 software package. Transmission electron microscopy (TEM) micrographies were performed on a JEOL 2100 FEG S/TEM microscope operated at 200 kV and equipped with a spherical aberration probe corrector. Before analysis, the samples were dispersed in ethanol and deposited on a holey carbon-coated TEM grid. In STEM, the images were recorded using a high-angular annular dark field (HAADF) detector with inner and outer diameters of about 73 and 194 mrad, respectively. Energy dispersive X-ray spectroscopy (EDS) analyses were performed in STEM using a JEOL silicon drift detector (DrySD60GV; sensor size, 60 mm^2) with a solid angle of approximately 0.6 srad. Particle size distribution and *d*-spacing calculation were carried out using ImageJ software. X-ray photoelectron spectroscopy (XPS) was carried out on a Kratos Axis Ultra spectrometer equipped with a mono-chromatized Al-Kα source (15 mA, 14 kV). C 1s core level (285.0 eV) was used as a reference to calibrate Binding Energy (BE) values. Data analysis was carried out using Casa XPS software and the Gaussian(70)-Lorentzian(30) line profile was adopted for the peak deconvolution.

Catalytic tests. For the hydrodeoxygenation reactions, a stainless-steel autoclave was filled with 10 mL of a solution 0.015 M of the carbonylic compound (0.15 mmol) in iPrOH, and catalyst (50 mg); then, the autoclave was sealed, and the reaction pressure (p = 20 bar) was subsequently generated using H_2. The reaction mixture was then stirred for 2 h at 600 rpm, at room temperature. After reaction completion, the reaction mixture is filtered and analyzed via gas chromatography (GC). In particular, an Agilent model 7890B chromatograph equipped with a flame ionization detector (GC-FID) coupled to an Agilent mass spectrometer (GC-MS), model 5977B, was used, allowing the quantification and identification of the products through the mass spectra interpretation of each compound. The chromatographic column (Agilent, CP-Wax 52 CB with 30 m length, 0.25 mm i.d., and 0.25 μm d_f) remained at 60 °C for 1 min, then it was heated at 10 °C min^{-1} until it reached 250 °C, and it remained at this temperature for 7 min. Helium was used as the carrier gas with a flow rate of 1.69 mL min^{-1}. For the HMF oxidation, indeed, a test tube was filled with 5 mL of a 0.01 M solution of HMF in water, along with catalyst (5 mg), and base (3 eq, for various bases), and subsequently sealed. The reaction mixture was then stirred for 4 h at 600 rpm, at the desired temperature. After reaction completion, the reaction mixture is filtered and analyzed via high-performance liquid chromatography (HPLC), using a ThermoScientific Vanquish chromatograph, equipped with a UV detector set at 265 nm. Samples (20 μL) were injected directly onto a Phenomenex ROA-Organic Acids

column at 30 °C in isocratic elution mode (0.6 mL min^{-1}), with a 5 mmol L^{-1} H_2SO_4 solution as the mobile phase. When conducting either analysis, 45 min of equilibration was required before the first sample injection. Starting material and products were analyzed separately to identify their retention times on the chromatogram; limiting reagent, intermediates, and product calibration curves were carried out, to calculate conversion, yields, and selectivity.

DFT calculations. DFT calculations were performed with the periodic VASP code,[1–3] by considering the generalized gradient approximation, as implemented in the Perdew–Burke–Ernzerhof (PBE) functional.[4] To account for the dispersion interactions we have included the Grimme's D3 parametrization.[5] The valence electrons have been expanded on a set of plane waves with a kinetic energy cutoff of 400 eV, whereas the core electrons were treated with the projector augmented wave approach (PAW) and by adopting the recommended pseudopotential from VASP.[6,7] The threshold criteria for electronic and ionic loops were set to 1×10^{-5} eV and 1×10^{-2} eV Å^{-1}, respectively. To simulate the hydrodeoxygenation of carbonylic compounds, first, we assumed that the reactions happen in two of the most abundant surfaces of palladium and platinum, (111) and (100) surfaces, and afterward, we considered the adsorption of four representative functional groups: acetic acid, acetone, ethanol, and propane onto these surfaces. To be more specific, we generated the following six periodic surfaces: Pd(111), Pt(111), $Pd_{0.90}Pt_{0.10}$(111), Pd(100), Pt(100), and $Pd_{0.87}Pt_{0.13}$(100). The solvent effect and the role of TiO_2 were not considered for the simulations. The surfaces were generated as follows, first, we fully optimized the palladium and platinum bulk parameters, afterward we used these optimized lattice parameters to generate a 2×2 supercell for Pd(111), and Pt(111) surfaces and a 3×3 supercell for Pd(100), and Pt(100) surfaces. The (111) surface had 5 layers with 16 atoms per plane while the (100) surface had 4 layers with 18 atoms per plane. On the other hand, for the 2×2 $Pd_{0.90}Pt_{0.10}$(111) supercell and 3×3 $Pd_{0.87}Pt_{0.13}$(100) supercell, the lattice parameters were approximated by the average of the DFT calculated bulk lattice parameters of the pure metals, where only the topmost layer was alloyed with 10% and ~13% of Pt in both the Pd(100) and Pd(111) surfaces. To avoid spurious effects between a periodic replica of the system along the non-periodic direction, the surfaces were periodically repeated with a vacuum of 20 Å, and for the (111) surfaces and the (100) surfaces, three and two bottom layers were frozen. During

the adsorption study on the pure surfaces, Pd(100), Pd(111), Pt(100), and Pt(111), only one adsorption site was considered. However, when we had alloyed surfaces, two or three adsorption sites were considered.

Table S1. Control experiments for the benzophenone hydrodeoxygenation over bimetallic Pt_xPd_y catalysts.

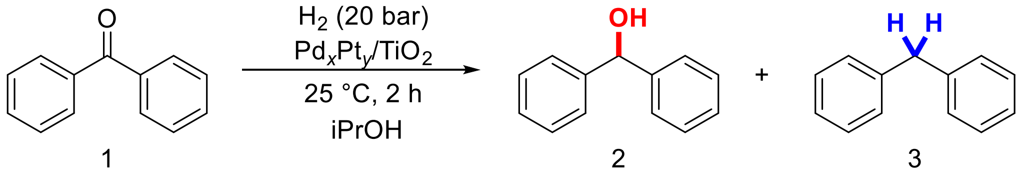

**Entry**	**Variation**	$X_{\text{benzophenone}}$ **(%)**	S_{HDO} **(%)**
1	No catalyst	0	0
2	TiO_2 as catalyst	0	0
3	20 bar N_2	0	0
4	No variation from the best conditions	100	73

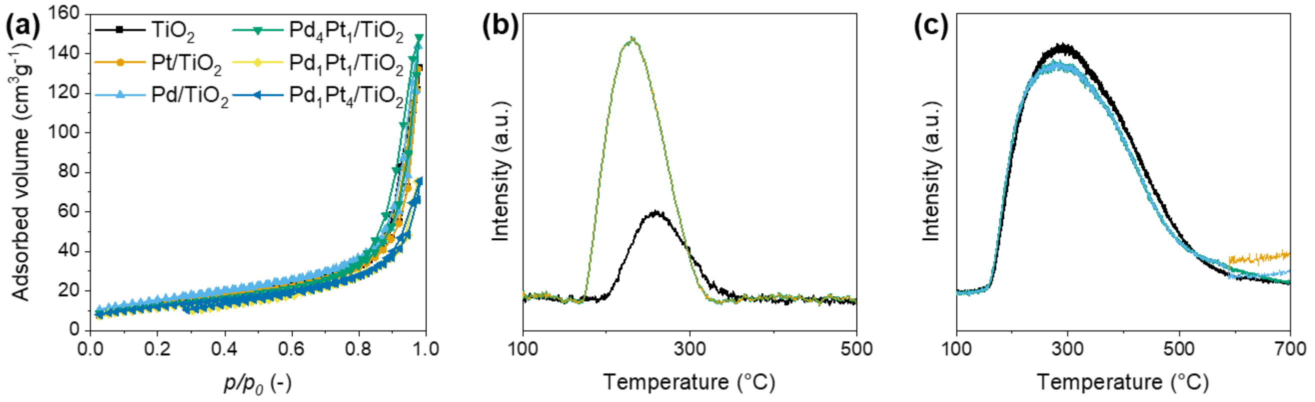

Figure S1. N_2 physisorption experiments (a) and pore size distribution (a, inset), CO_2-TPD (b), and NH_3-TPD curves for monometallic and bimetallic catalysts. The legend in (a) applies also to (b) and (c).

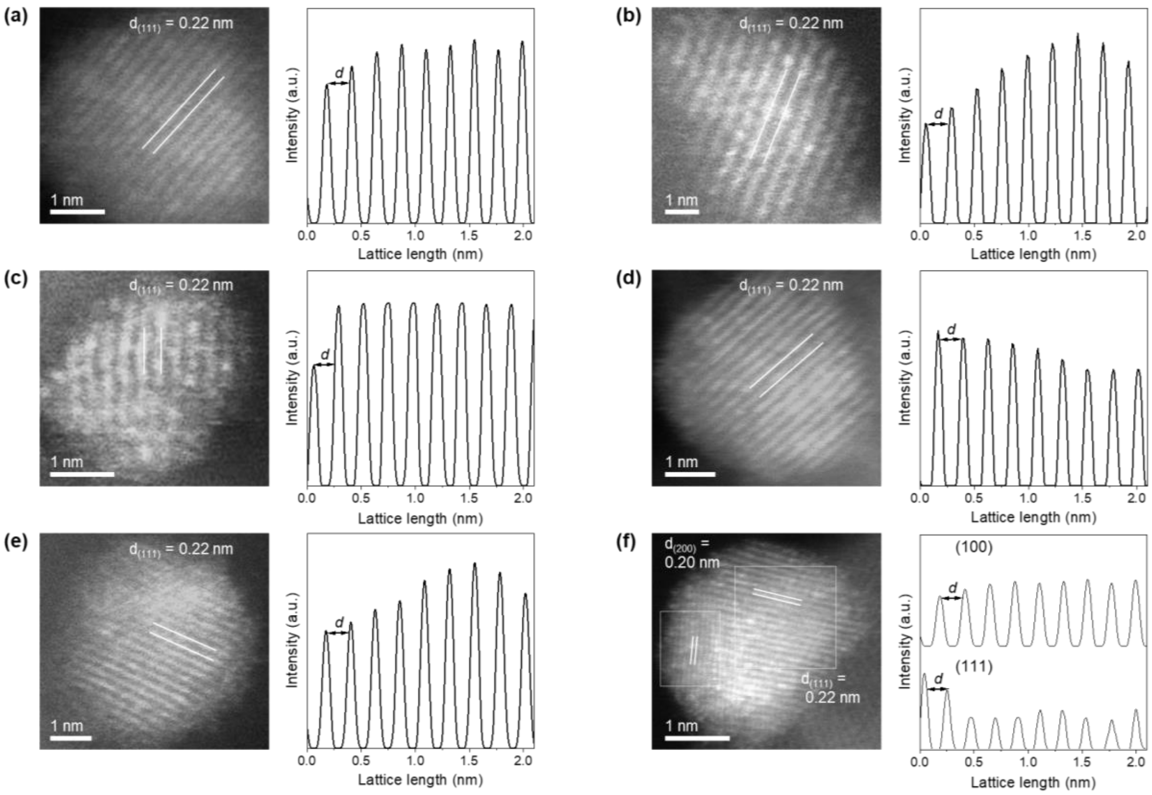

Figure S2. Crystalline facets identification obtained through HRTEM lattice analysis and *d*-spacing calculations in several regions of bimetallic samples.

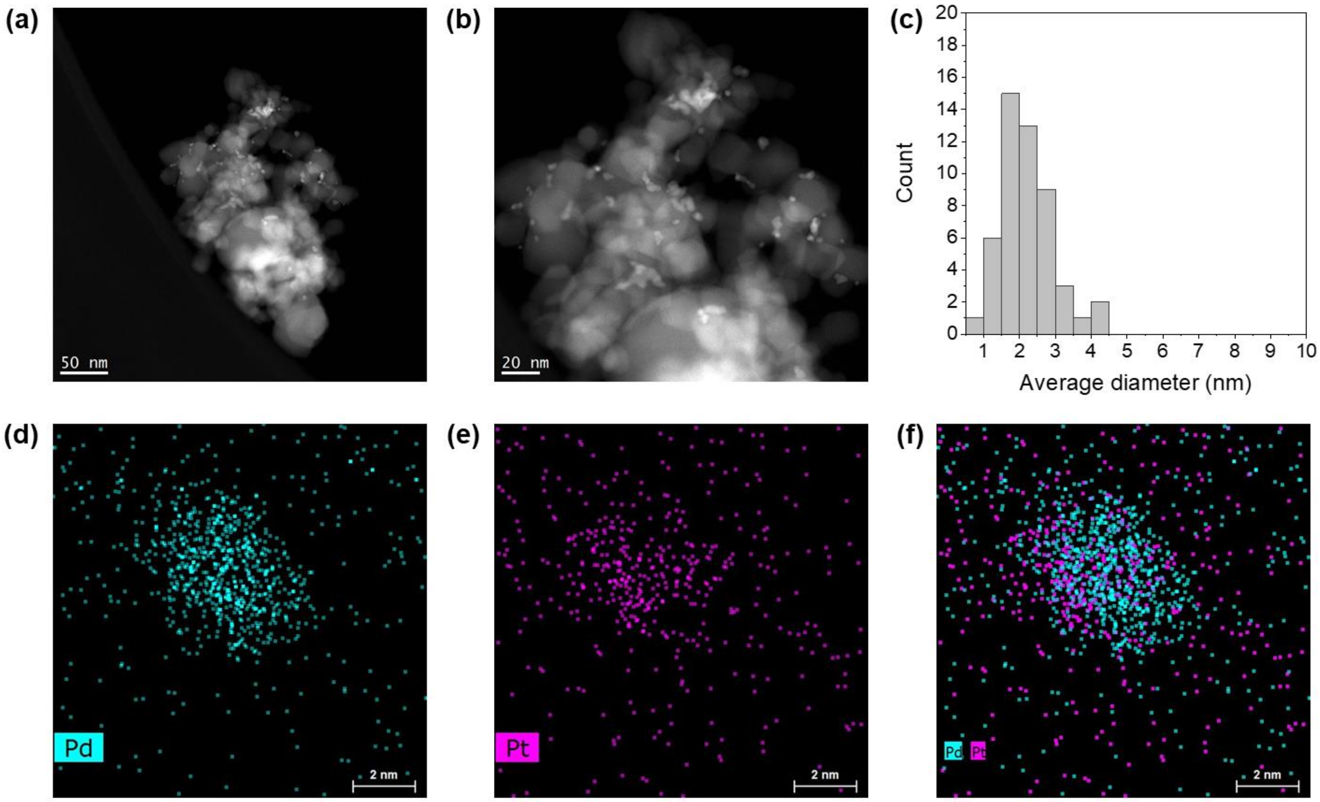

Figure S3. TEM characterization of the Pd_4Pt_1/TiO_2 catalyst after five recyclability tests following benzophenone hydrodeoxygenation reactions. (a,b) show high-resolution TEM images highlighting the morphology and particle dispersion; (c) provides the particle size distribution analysis; (d,e) display elemental mapping of palladium and platinum, respectively; and (f) presents the superimposed elemental maps, confirming the uniform distribution and bimetallic nature of the catalyst.

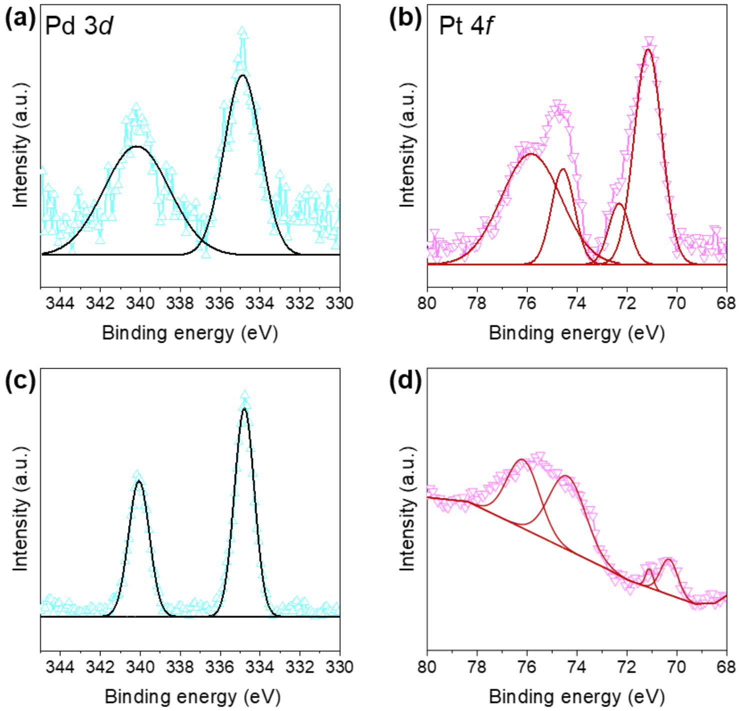

Figure S4. XPS spectra of Pd (a) and Pt (b) for the fresh Pd_4Pt_1/TiO_2 catalyst, along with a comparison of the XPS spectra of the same catalyst after five recyclability tests following benzophenone hydrodeoxygenation reactions (c and d). For the reaction conditions, see Figure 3e in the main manuscript.

DFT results

Pd(111). The top view, side view, and the adsorption energy for each molecule on the Pd(111) surface are reported in **Figure S5**, while the bond distances between the adsorbate and adsorbent are summarized in **Table S2**. It is important to mention that we have considered two different configurations for the propane molecule, where Propane I forms the more stable intermediate. In all the cases, only one Pd adsorption site was analyzed.

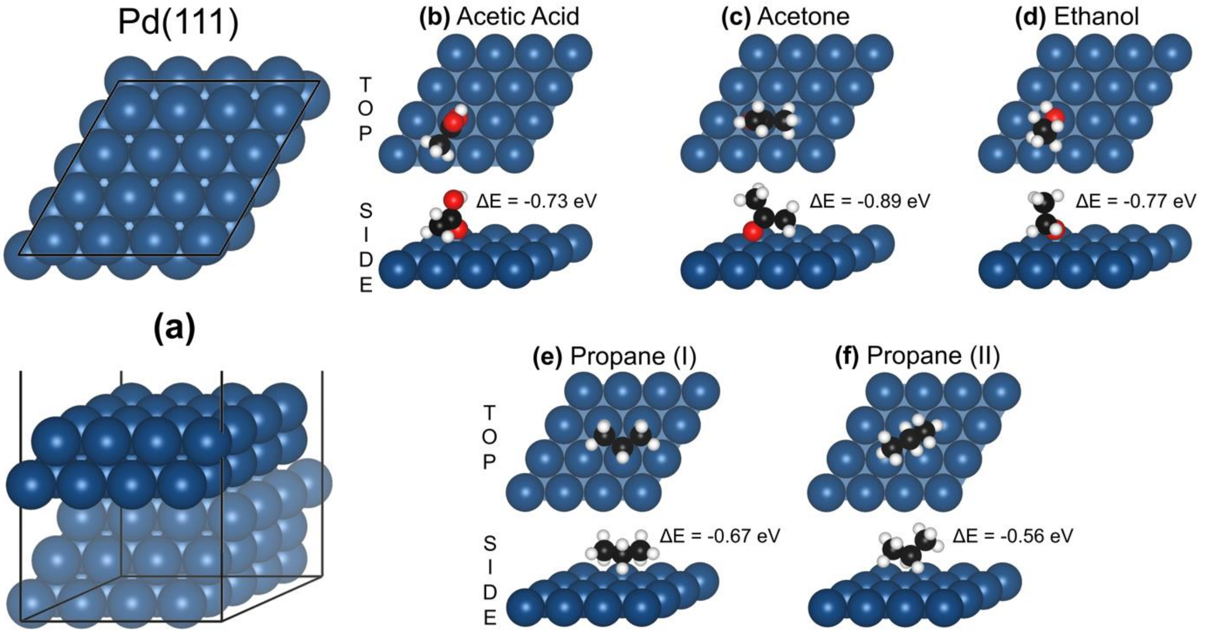

Figure S5. Top and side view of the Pd(111) surface (a). The top and side views for each functional group are shown for acetic acid (b), for acetone (c), for ethanol (d), and for propanol (e-f). The blue, black, red, and white balls represent the palladium, carbon, oxygen, and hydrogen atoms.

Table S2. Bond distances between the adsorbate and adsorbent on the Pd(111) surface.

Adsorbate	Pd-O (Å)	Pd-C (Å)	Pd-H (Å)	O-C (Å)	C-O (Å)	C-C (Å)
Acetic acid	2.26	3.15	2.57	1.24	1.35	1.49
Acetone	2.20	3.17	2.62	1.25	-	1.49-1.50
Ethanol	2.26	3.19	2.78	1.46	-	1.51
Propane I	-	3.21	2.21	-	-	1.52
Propane II	-	3.00	2.13	-	-	1.52

Pt(111). The binding energies for each functional group on the Pt(111) surface are reported in **Figure S6**, and in **Table S3**, we summarized the bond distance between the adsorbate and adsorbent. For this surface, two different configurations for the propane molecule on Pt(111) were studied, and even for this surface, Propane I forms the more stable intermediate.

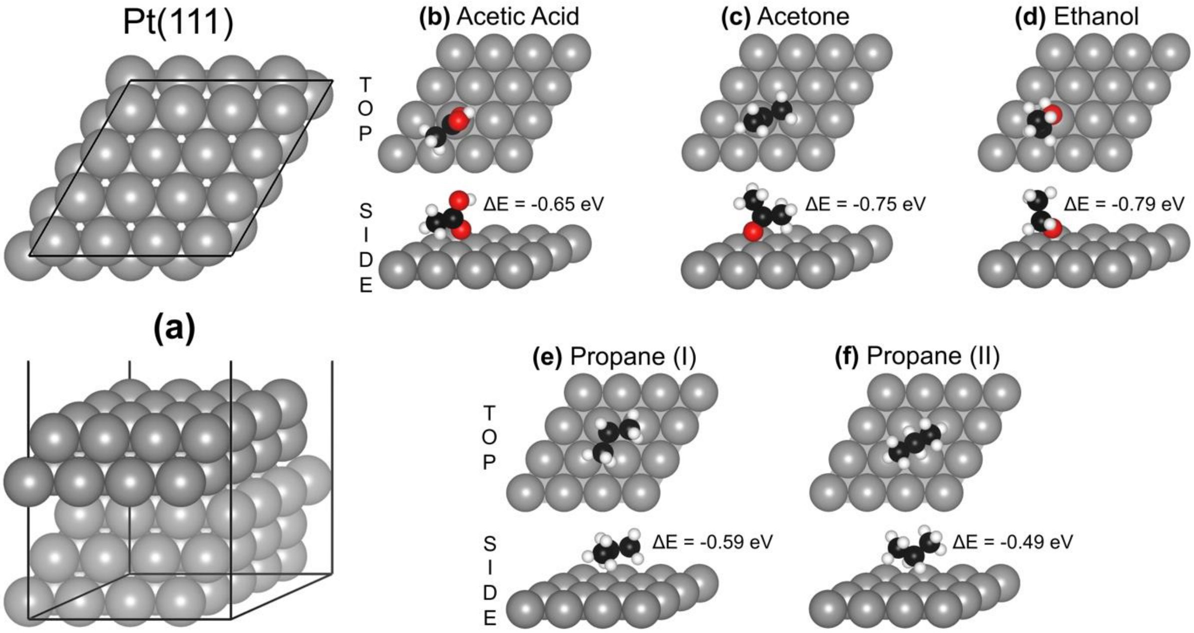

Figure S6. Top and side view of the Pt(111) surface (a). The top and side views for each functional group are shown for acetic acid (b), for acetone (c), for ethanol (d), and for propanol (e-f). The grey, black, red, and white balls represent the platinum, carbon, oxygen, and hydrogen atoms.

Table S3. Bond distances between the adsorbate and adsorbent on the Pt(111) surface.

Adsorbate	Pd-O (Å)	Pd-C (Å)	Pd-H (Å)	O-C (Å)	C-O (Å)	C-C (Å)
Acetic acid	2.27	3.21	2.62	1.24	1.34	1.48
Acetone	2.21	3.22	2.52	1.25	-	1.49-1.50
Ethanol	2.28	3.25	2.58	1.46	-	1.51
Propane I	-	3.29	2.20	-	-	1.52
Propane II	-	3.30	2.23	-	-	1.52

$Pd_{0.90}Pt_{0.10}$(111). The adsorption energy for each molecule on the $Pd_{0.90}Pt_{0.10}$(111) surface is reported in **Figure S7**, and in **Table S4**, we are reporting the bond distance between the adsorbate and adsorbent. In this case, only the topmost layer of the surface is alloyed, therefore, we have considered two binding sites for each molecule, Pt (site I) or Pd (site II), except for propane, where we considered three sites.

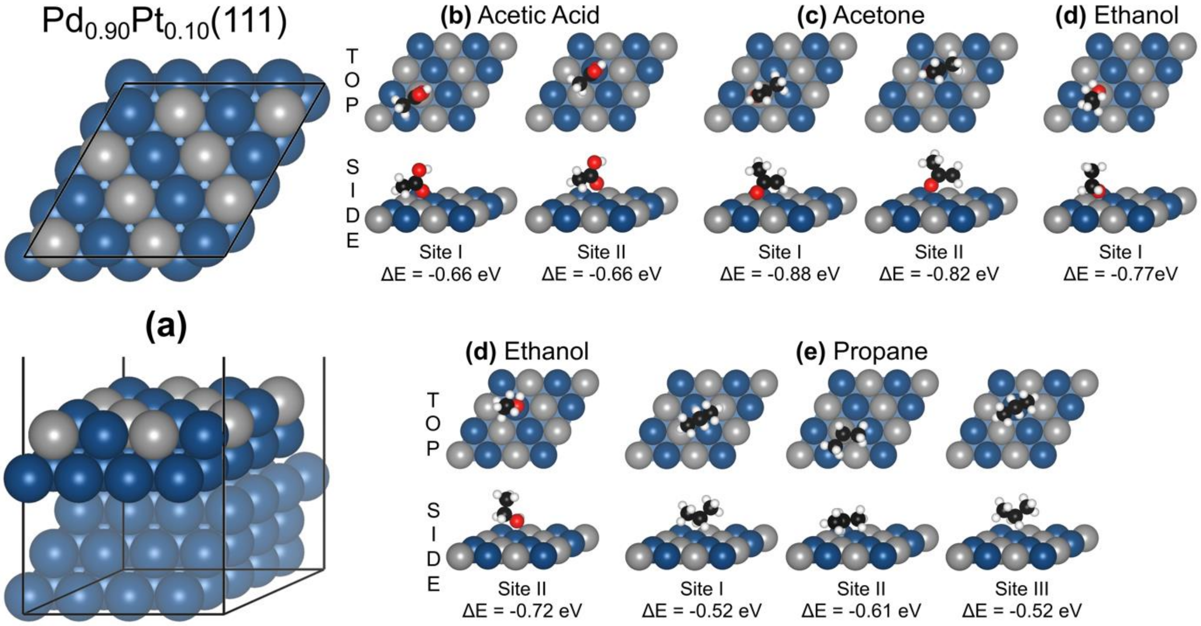

Figure S7. Top and side view of the $Pd_{0.90}Pt_{0.10}$(111) surface (a). The top and side views for each functional group in the different binding sites are shown in for acetic acid (b), for acetone (c), for ethanol (d), and for propanol (e). The grey, blue, black, red, and white balls represent the platinum, palladium, carbon, oxygen, and hydrogen atoms.

Table S4. Bond distances between the adsorbate and adsorbent on the $Pd_{0.90}Pt_{0.10}(111)$ surface.

Adsorbate	Pd-O (Å)	Pd-C (Å)	Pd-H (Å)	O-C (Å)	C-O (Å)	C-C (Å)
Acetic acid I[a]	2.24	3.21	2.47	1.24	1.34	1.48
Acetic acid I[a]	2.31	3.28	2.57	1.24	1.35	1.49
Acetone I	2.16	3.14	2.35	1.25	-	1.48-1.50
Acetone II	2.23	3.21	2.49	1.24	-	1.49-1.50
Ethanol I	2.24	3.19	2.55	1.46	-	1.51
Ethanol II	2.28	3.22	2.62	1.46	-	1.51
Propane I	-	3.17	2.27	-	-	1.52
Propane II	-	3.15	2.06	-	-	1.52
Propane III	-	2.84	2.28	-	-	1.52

[a]Within the acetic acid molecule, both sites have the same binding energy, -0.66 eV, while for acetone and ethanol, the molecules adsorb better in the Pt site (site I).

Pd(100). The binding energy for each adsorbed species on the Pd(100) surface is reported in **Figure S7**, while in **Table S5**, we summarized the bond distance between the adsorbate and adsorbent. As for the Pd(111) surface, only one adsorption site was considered for the four functional groups.

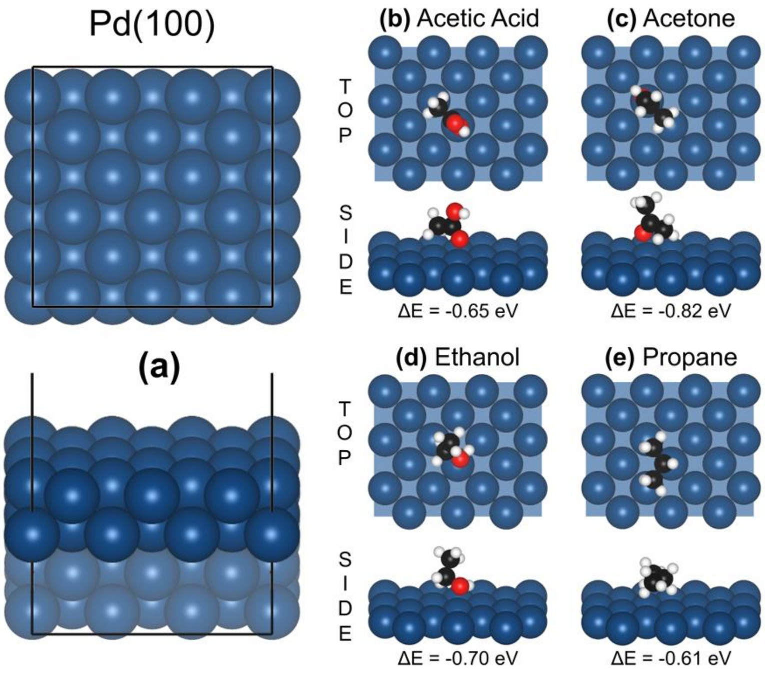

Figure S8. Top and side view of the Pd(100) surface (a). The top and side views for each functional group are shown for acetic acid (b), for acetone (c), for ethanol (d), and for propanol (e). The blue, black, red, and white balls represent the palladium, carbon, oxygen, and hydrogen atoms.

Table S5. Bond distances between the adsorbate and adsorbent on the Pd(100) surface.

**Adsorbate**	**Pd-O (Å)**	**Pd-C (Å)**	**Pd-H (Å)**	**O-C (Å)**	**C-O (Å)**	**C-C (Å)**
Acetic acid	2.27	3.13	2.58	1.24	1.35	1.49
Acetone	2.16	3.09	2.57	1.25	-	1.49-1.50
Ethanol	2.30	3.21	2.29	1.45	-	1.51
Propane	-	3.16	2.35	-	-	1.52

Pt(100). As with Pd(100), the adsorption energy for each functional group on the Pt(100) surface is reported in **Figure S9**, and in **Table S6**, the bond distances between the adsorbate and adsorbent are reported.

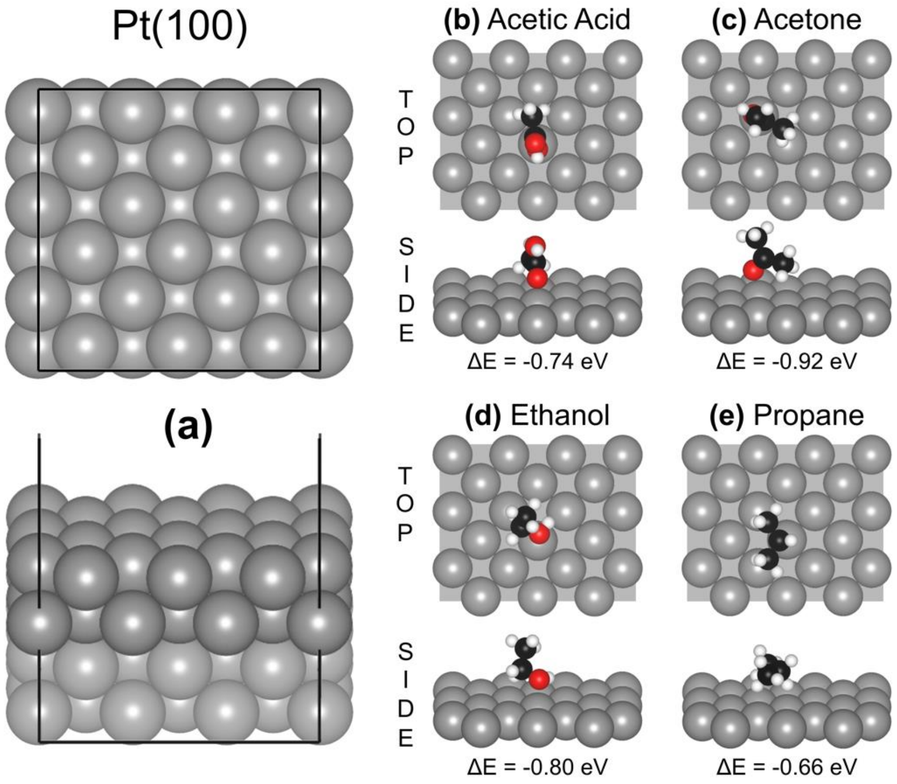

Figure S9. Top and side view of the Pt(100) surface (a). The top and side views for each functional group are shown for acetic acid (b), for acetone (c), for ethanol (d), and for propanol (e). The blue, black, red, and white balls represent the palladium, carbon, oxygen, and hydrogen atoms.

Table S6. Bond distances between the adsorbate and adsorbent for the Pt(100) surface.

**Adsorbate**	**Pd-O (Å)**	**Pd-C (Å)**	**Pd-H (Å)**	**O-C (Å)**	**C-O (Å)**	**C-C (Å)**
Acetic acid	2.25	3.18	2.54	1.24	1.34	1.48
Acetone	2.18	3.13	2.35	1.25	-	1.48-1.50
Ethanol	2.30	3.21	2.28	1.45	-	1.51
Propane	-	3.15	2.11	-	-	1.52

$Pd_{0.87}Pt_{0.13}$(100). The binding energies for each molecule on the $Pd_{0.87}Pt_{0.13}$(100) surface are reported in **Figure S10**, and in **Table S7**, the bond distances between the adsorbate and adsorbent are summarized. As we now have an alloy in the topmost layer, we have considered two binding sites for each functional group, even for the propane molecule.

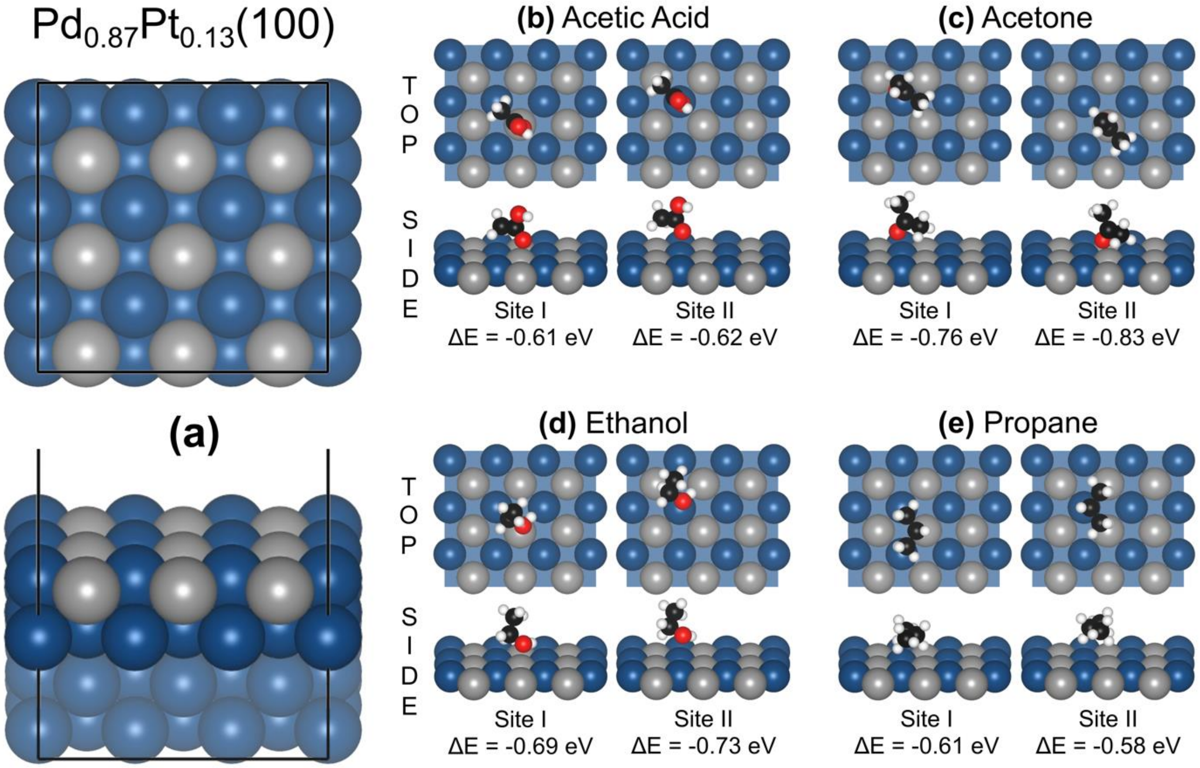

Figure S10. Top and side view of the $Pd_{0.87}Pt_{0.13}$(100) surface (a). The top and side views for each functional group in the different binding sites are shown for acetic acid (b), for acetone (c), for ethanol (d), and for propanol (e). The blue, black, red, and white balls represent the palladium, carbon, oxygen, and hydrogen atoms.

Table S7. Bond distances between the adsorbate and adsorbent on $Pd_{0.87}Pt_{0.13}(100)$ surface.

Adsorbate	Pd-O (Å)	Pd-C (Å)	Pd-H (Å)	O-C (Å)	C-O (Å)	C-C (Å)
Acetic acid I	2.21	3.14	2.54	1.25	1.34	1.48
Acetic acid I	2.33	3.28	2.57	1.24	1.35	1.49
Acetone I	2.11	3.05	2.61	1.26	-	1.48-1.49
Acetone II	2.22	3.16	2.54	1.24	-	1.49-1.50
Ethanol I	2.25	3.19	2.29	1.46	-	1.51
Ethanol II	2.37	3.24	2.25	1.45	-	1.51
Propane I	-	3.18	2.25	-	-	1.52
Propane II	-	3.17	2.18	-	-	1.52

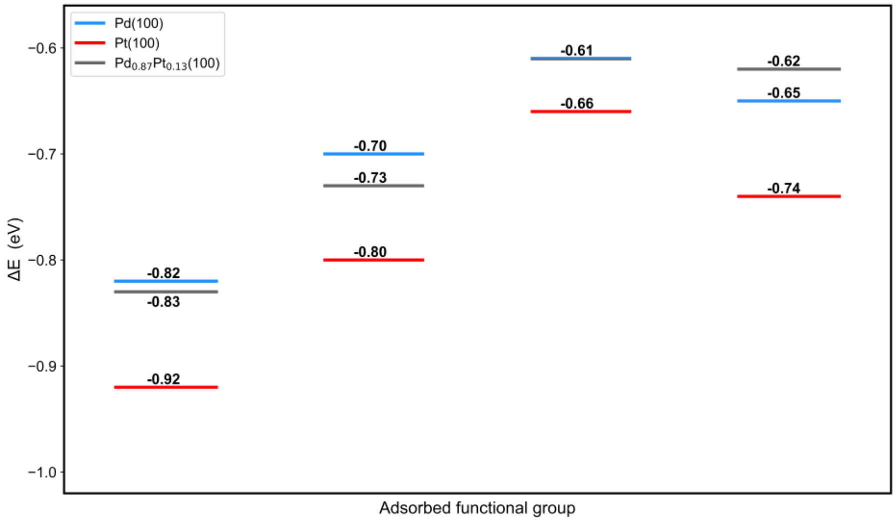

Figure S11. Adsorption binding energies for each functional group on the different Pd(100), Pt(100), and $Pd_{0.87}Pt_{0.13}(100)$ surfaces for monometallic and bimetallic catalysts. From left to right: carbonyl, hydroxyl, alkene, and carboxyl functional groups.

Table S8. Optimization of the reaction conditions for the HMF oxidation over bimetallic Pt_xPd_y catalysts.

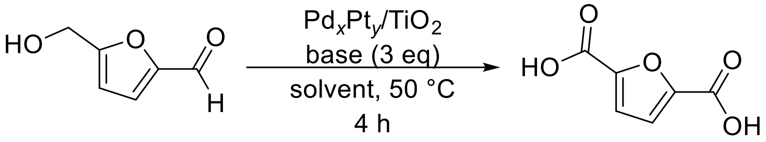

Entry	Catalyst	Catalyst amount	Base	Solvent	Reaction rate ($mmol_{prod}\ g_{cat}^{-1}\ h^{-1}$)
1	Pd_4Pt_1/TiO_2	5 mg	CaF_2	H_2O	0
2	Pd_4Pt_1/TiO_2	5 mg	K_2CO_3	H_2O	0.16
3	Pd_4Pt_1/TiO_2	5 mg	NaOH	H_2O	0.34
4	Pd_4Pt_1/TiO_2	5 mg	NaOH	MeCN	0
5	Pd_4Pt_1/TiO_2	5 mg	NaOH	iPrOH	0.11
6	Pd_4Pt_1/TiO_2	2.5 mg	NaOH	H_2O	0.32
7	Pd_4Pt_1/TiO_2	10 mg	NaOH	H_2O	0.05

Table S9. Control experiments for the HMF oxidation over bimetallic Pt_xPd_y catalysts.

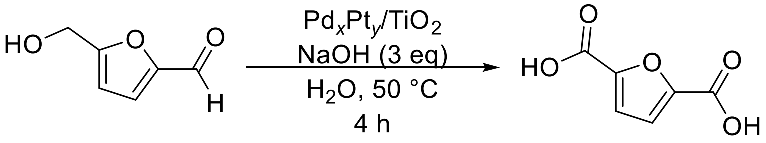

Entry	Variation	Reaction rate ($mmol_{prod}\ g_{cat}^{-1}\ h^{-1}$)
1	No base	0
2	No catalyst	0
3	TiO_2 as catalyst	0
4	No variation from the best conditions	0.34

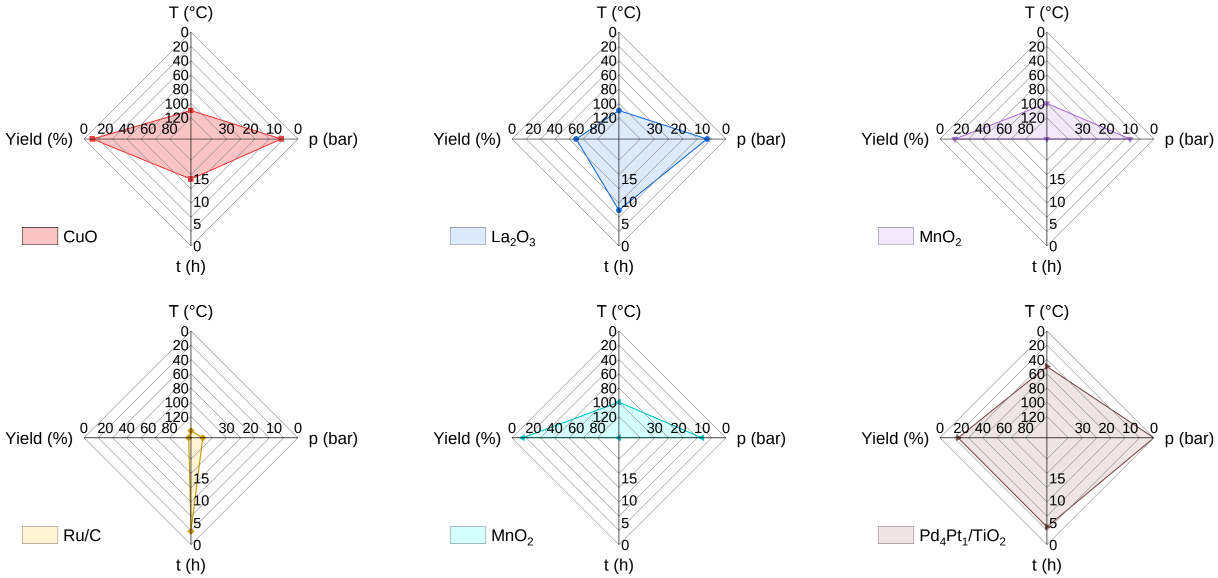

Figure S12. Literature precedents for the oxidation of HMF over various heterogeneous catalysts. References for the catalysts: CuO [8], La_2O_3 [9]; MnO_2 [10]; Ru/C [11]; MnO_2 [12]; Pd_4Pt_1/TiO_2 this work.

References of the Supporting Information

1 G. Kresse, J. Hafner, *Phys. Rev. B* **1993**, *47*, 558-561.

2 G. Kresse, J. Hafner, *Phys. Rev. B* **1994**, *49*, 14251-14269.

3 G. Kresse, J. Furthmüller, *Comput. Mater. Sci.* **1996**, *6*, 15-50.

4 J. P. Perdew, K. Burke, M. Ernzerhof, *Phys. Rev. Lett.* **1996**, *77*, 3865-3868.

5 S. Grimme, J. Antony, S. Ehrlich, H. Krieg, *J. Chem. Phys.* **2010**, *132*, 154104.

6 P. E. Blöchl, *Phys. Rev. B* **1994**, *50*, 17953-17979.

7 G. Kresse, D. Joubert, *Phys. Rev. B* **1999**, *59*, 1758-1775.

8 M. Ventura, M. Aresta, A. Dibenedetto, *ChemSusChem* **2016**, *9*, 1096-1100.

9 L. Zheng, J. Zhao, Z. Du, B. Zong, H. Liu, *Sci. China Chem.* **2017**, *60*, 950-957.

10 E. Hayashi, T. Komanoya, K. Kamata, M. Hara, *ChemSusChem* **2017**, *10*, 654-658.

11 W. Xie, H. Liu, X. Tang, X. Zeng, Y. Sun, X. Ke, T. Li, H. Fang, L. Lin, *Appl. Catal. A* **2022**, *630*, 118463.

12 M. Mani, M. Mariyaselvakumar, S. Tothadi, A. B. Panda, K. Srinivasan, L. J. Konwar, *Mol. Catal.* **2024**, *554*, 113811.
